# Effect on Cognitive Behavioral Therapy Combined With Transcranial Direct Current Stimulation on Anxiety and Depression in Stroke Patients With Dysphagia

**DOI:** 10.62641/aep.v54i3.2204

**Published:** 2026-06-15

**Authors:** Wenlin Sun, Yangyang Liu, Chang Xu, Huishan Zhu

**Affiliations:** ^1^Department of Rehabilitation Medicine, The Affiliated Huaian No.1 People’s Hospital of Nanjing Medical University, 223300 Huaian, Jiangsu, China

**Keywords:** transcranial direct current stimulation, cognitive behavioural therapy, Stroke, Dysphagia, anxiety, depression

## Abstract

**Background:**

This study aimed to investigate the association between cognitive behavioural therapy (CBT) combined with transcranial direct current stimulation (tDCS) on negative mood states, such as anxiety and depression, in patients with post-stroke dysphagia through a retrospective review of medical records.

**Methods:**

A retrospective study was conducted using medical records of patients with post-stroke dysphagia who were hospitalised at The Affiliated Huaian No. 1 People’s Hospital of Nanjing Medical University between January 2022 and June 2025. Sample size matching was performed using the nearest neighbour matching method in propensity score matching on the basis of data extracted from medical records, ultimately including 348 study subjects for analysis. Based on the treatment regimens documented in the medical records, 174 patients in the combination group received CBT combined with tDCS, and 174 patients in the control group received tDCS alone. Basic rehabilitation measures were consistent between the two groups as per clinical protocols. Both groups completed a continuous 4-week treatment course as recorded in the medical charts. Data on the grading of the water swallowing test, surface electromyography parameters, the Stigma Scale for Chronic Illness (SSCI) score, the Herth Hope Index (HHI) score, the Self-Rating Anxiety Scale (SAS) score and the Self-Rating Depression Scale (SDS) score were extracted from the medical records at baseline (within 48 h before treatment initiation) and after the 4-week treatment period.

**Results:**

Following the 4-week treatment period, the overall distribution of water swallowing test grades documented in the medical records for the combination group was superior to that for the control group (*p* < 0.05). At baseline, no statistically significant differences were found in the surface electromyography parameters (average and maximum amplitudes) retrieved from the records between the two groups (*p* > 0.05). After the 4-week treatment period, the recorded values for average and maximum amplitudes in the combination group were significantly higher than those in the control group (*p* < 0.05). At baseline, no statistically significant differences were observed in various stigma scale scores extracted from the records between the two groups (*p* > 0.05). After the treatment period, the scores for internalised stigma and the total SSCI score in the combination group were significantly lower than those in the control group (*p* < 0.05). At baseline, no statistically significant differences were noted in any dimension scores of HHI between the two groups (*p* > 0.05). After the treatment period, the scores for positive attitude toward reality and the future, positive actions taken and maintaining close relationships with others and the total HHI score were all markedly higher in the combination group than in the control group (*p* < 0.05). At baseline, no statistically significant differences were observed in the SAS and SDS scores between the two groups (*p* > 0.05). After the treatment period, the SAS and SDS scores in the combination group were evidently lower than those in the control group (*p* < 0.05).

**Conclusions:**

The combination of CBT and tDCS was associated with improved swallowing function; reduced stigma, depression and anxiety; and increased hope levels in patients with post-stroke dysphagia.

## Introduction

Stroke is characterised by high incidence, high disability rates and high recurrence rates, with ischemic stroke accounting for the highest proportion in clinical practice [1]. Dysphagia is a common complication following ischemic stroke. It not only leads to malnutrition, dehydration and aspiration pneumonia, which considerably impair patients’ quality of life, but also causes substantial psychological distress due to the loss of the basic physiological and social function of eating [2]. Post-stroke dysphagia is frequently accompanied by symptoms of anxiety and depression, which may further impair patients’ quality of life and rehabilitation outcomes [3]. These negative psychological states are associated with reduced rehabilitation adherence and increased physical and psychological burden [4]. Currently, the clinical treatment for post-stroke dysphagia primarily relies on non-invasive brain neuromodulation techniques such as transcranial direct current stimulation (tDCS), along with conventional swallowing training, with a focus on the remodelling of neural pathways and the restoration of muscle function [5]. However, purely physical rehabilitation often overlooks the internal psychological reconstruction of patients. Cognitive behavioural therapy (CBT) has been shown in a systematic review to be effective in reducing hopelessness and enhancing hope, particularly in older adults with depressive symptoms [6]. However, the efficacy of CBT in alleviating anxiety and depression, specifically in patients with post-stroke dysphagia, has not been fully established. Existing research has predominantly focused on single-modality neurological function modulation, with a notable lack of comprehensive studies that integrate physical therapy with in-depth psychological interventions to synergistically address the severe stigma and low hope levels in patients with dysphagia following ischemic stroke. The present study identified patients with dysphagia after ischemic stroke from medical records to investigate the association between CBT combined with tDCS and negative mood changes such as anxiety and depression.

## Materials and Methods

### General Data

This study was a retrospective cohort study. Patients with post-stroke dysphagia who were hospitalised at The Affiliated Huaian No. 1 People’s Hospital of Nanjing Medical University from January 2022 to June 2025 were identified from the hospital’s electronic medical record system as the source population. The diagnostic criteria for patients with stroke referred to the standards in the “Chinese Guidelines for the Diagnosis and Treatment of Acute Ischemic Stroke 2018” [7], and all cases had been confirmed by cranial CT and MRI examinations as documented in the records. The inclusion criteria were as follows: (1) inpatients aged ≥ 50 years considering that the common age of onset for patients with stroke is mainly over 50 years, (2) diagnosis of post-stroke dysphagia confirmed by videofluoroscopic swallowing study [8] and water swallowing test [9], (3) conscious and in stable condition according to admission and progress notes and (4) duration of post-stroke dysphagia > 2 weeks. The exclusion criteria were as follows: (1) pre-existing dysphagia before stroke, as documented in past medical history; (2) history of oesophageal tumours or previous trauma or surgery; (3) record of impaired consciousness; (4) haemorrhagic cerebrovascular disease; (5) neuromuscular diseases; and (6) intracranial infection, trauma or space-occupying lesions. The specific identification and matching process was as follows: A total of 716 patients with post-stroke dysphagia were initially identified during the study period. On the basis of the pre-set inclusion and exclusion criteria applied to the medical records, 264 patients were excluded. After the exclusion criteria were applied, a total of 452 patients met the eligibility criteria based on medical record review and constituted the pre-matching sample size. According to the treatment regimens documented in the medical records, 239 patients received CBT combined with tDCS, and 213 patients received tDCS alone. This study was approved by the Medical Ethics Committee of The Affiliated Huaian No.1 People’s Hospital of Nanjing Medical University (approval number: KY-2026-018-01).

Propensity score matching (PSM) process: Matching objective: This study employed PSM by using data extracted from medical records to reduce confounding bias and categorise patients into the combination group (CBT + tDCS) and the control group (tDCS alone). Matching factors: Potential confounding factors that may influence treatment assignment were selected as covariates to construct a logistic regression model for calculating propensity scores. The specific matching variables included age, sex, body mass index (BMI), National Institutes of Health Stroke Scale (NIHSS) score, years of education, duration of dysphagia, cerebral infarction classification (anterior/posterior circulation), smoking history, alcohol consumption history, comorbidities (hypertension, diabetes and hyperlipidaemia) and use of antipsychotic medication. The nearest neighbour matching method was used, with a calliper width set at 0.2 to ensure matching quality. Matching result: Amongst the 452 patients, 174 pairs (combination and control groups) were successfully matched, resulting in a final study population of 348 patients. The two groups were balanced and comparable in terms of matching factors (*p* > 0.05), as detailed in Table 1.

**Table 1.** **Comparison of general characteristics between the combination and control groups**.

General data		Combination group (n = 174)	Control group (n = 174)	t/χ^2^	*p*
Age (years old)		71.43 ± 7.44	70.91 ± 6.92	0.675	0.500
BMI (kg/m^2^)		23.98 ± 1.74	23.77 ± 1.94	1.063	0.289
NIHSS score (point)		8.37 ± 1.88	8.45 ± 1.86	–0.399	0.690
Years of education (years)		5.92 ± 1.59	5.97 ± 1.53	–0.299	0.765
Duration of dysphagia (d)		18.70 ± 1.49	18.48 ± 1.55	1.360	0.175
Gender (%)				0.942	0.332
	Female	82 (47.13)	73 (41.95)		
	Male	92 (52.87)	101 (58.05)		
Classification of cerebral infarction (%)				0.196	0.658
	Anterior circulation	63 (36.21)	67 (38.51)		
	Posterior circulation	111 (63.79)	107 (61.49)		
Smoking (%)				1.236	0.266
	Yes	59 (33.91)	69 (39.66)		
	No	115 (66.09)	105 (60.34)		
Drinking (%)				1.817	0.178
	Yes	55 (31.61)	67 (38.51)		
	No	119 (68.39)	107 (61.49)		
Types of comorbidities (%)					
	Hypertension	143 (82.18)	134 (77.01)	1.433	0.431
	Diabetes	43 (24.71)	54 (31.03)	1.729	0.188
	Hyperlipidaemia	146 (83.91)	157 (90.23)	3.088	0.079
Antipsychotic Medication					
	Escitalopram	21 (12.07)	25 (14.37)	0.401	0.527
	Sertraline	14 (8.05)	17 (9.77)	0.319	0.572

Note: BMI, body mass index; NIHSS, National Institutes of Health Stroke Scale.

### Basic Treatment and tDCS Therapy

Based on data extracted from the medication administration records, both groups received basic pharmacological treatment for secondary stroke prevention, which included antiplatelet aggregation drugs (such as Aspirin (100mg, Nanjing Baijingyu Pharmaceutical Co., Ltd., Batch No.: 202504211, Nanjing, China) or Clopidogrel Aspirin (75 mg/100 mg, Lepu Pharmaceutical Co., Ltd., Batch No.: A250205M1, Zhoukou, Henan, China)) based on aetiology, statin lipid-lowering drugs (such as atorvastatin calcium tablets (20mg, Qilu Pharmaceutical (Hainan) Co., Ltd., Batch No.: KCRH5415, Haikou, Hainan, China)) and medications for blood pressure and blood glucose control. For patients with documented anxiety and depressive symptoms upon admission, conventional anti-anxiety and antidepressant drugs, such as flexible doses of selective serotonin reuptake inhibitors (e.g., escitalopram 10–20 mg/d (Sichuan Kelun Pharmaceutical Co., Ltd., Batch No.: B251204H97, Sichuan, China)) [10], were administered under the guidance of psychiatric consultation. The medication regimens and dose adjustment principles were noted to be consistent between the two groups.

Both groups received tDCS in addition to their basic pharmacological treatment. According to the device usage logs, the MBM-Ⅳ600 Intelligent Electrical Stimulator (Jiangxi Huahuajingxing Medical Technology Co., Ltd., Medical Device Registration Certificate No. Gan Xie Zhun 20212090101) was used, with the stimulation mode set to direct current. Sponge electrodes (approximately 5 cm × 7 cm in area) were used. Prior to electrode placement, the skin was cleaned with alcohol pads to reduce impedance. Electrode placement followed the international 10-20 electroencephalography positioning system [11]. The anode was positioned at the C3 or C4 site over the swallowing-related motor cortex on the affected hemisphere (C3 for left hemisphere lesions and C4 for right hemisphere lesions). The cathode was placed on the contralateral supraorbital area. The stimulation intensity was set at 1.8 mA (finely adjusted within a range of 1.5–2.0 mA based on patient tolerance). Each stimulation session lasted 20 min, with 15 s ramp-up and -down periods at the beginning and end to minimise patient discomfort [12]. The treatment frequency was once daily, five times per week, for a continuous period of 4 weeks. Nursing records indicated that throughout the treatment, vital signs and subjective reactions were monitored by dedicated personnel. If patients experienced significant headache, dizziness, skin burning pain or other intolerable symptoms, the stimulation was immediately terminated, and adverse reactions were recorded.

### CBT

According to the medical and rehabilitation records, the patients in the combination group received a structured CBT protocol. The details are as follows:

(1) CBT was conducted during the enhanced neuroplasticity window. It was administered within 30 min after each tDCS session, three times per week, for a total of 12 sessions over 4 consecutive weeks. Each session lasted 40–45 min. The therapy was delivered by trained rehabilitation therapists or psychiatric specialist nurses who attended weekly group supervision from a chief psychiatrist to ensure technical consistency.

(2) In the initial first and second sessions of the treatment phase, the focus was establishing a therapeutic alliance and providing psychoeducation about the illness. During these sessions, motivational interviewing techniques were used to understand the patients’ perceptions and emotional responses regarding dysphagia. Therapists explained the neurological mechanisms underlying post-ischemic stroke swallowing impairment and the rehabilitation timeline to the patients. A key emphasis was placed on introducing the four-component recording method “situation-thought-emotion-discomfort level” to help patients recognise the vicious cycle between catastrophic thoughts (e.g., coughing whilst eating means choking) and their accompanying anxiety. Therapists collaborated with the patients to complete daily self-help records as homework, thereby gathering materials for the subsequent cognitive restructuring.

(3) Sessions 3–5 marked the entry into the swallowing-specific cognitive restructuring phase. Therapists used Socratic questioning to guide the patients in examining the validity of their negative automatic thoughts. For instance, addressing the stigmatising belief that “being fed by others makes me useless”, therapists facilitated cognitive defusion by helping the patients recall past experiences of competence, thereby separating the concept of “my function” from “my self”. Regarding eating-related fears, the patients were guided to compare their water swallowing test results with daily training records. This process replaced their original catastrophic cognitions with realistic evidence such as “coughing is a protective reflex” and “I can already safely swallow semi-liquid foods”. Concurrently, a graduated behavioural experiment was designed. The patients began by attempting small amounts of pureed food in the presence of their therapist and gradually progressed to social scenarios involving shared meals with family members. This step-by-step approach helped the patients verify the discrepancy between their actual social experiences and their pre-existing fears.

(4) Sessions 6–8 during the mid-treatment phase integrated behavioural activation with swallowing rehabilitation training. The therapist collaborated with the patients to develop a personalised swallowing fear hierarchy, using the water swallowing test grades as a reference for determining the intensity and difficulty of exposure exercises and breaking down goals into actionable weekly tasks. For example, for a patient at grade II, the weekly goal was set as “complete swallowing of 30 mL of warm water under therapist supervision, with no more than two swallows and no aspiration”. The achievement of weekly tasks was assessed on the basis of daily training records (e.g., if the final evaluation of the training met the above criteria, the week’s goal was considered achieved). The water swallowing test was not used as a repetitive training process during this phase. If aspiration or considerable discomfort occurred during training, the drinking exposure was immediately halted, and the difficulty level was reduced. Training resumed at a lower intensity once symptoms stabilised before gradually progressing again. Through the use of an activity scheduling table, the patients were encouraged to engage in at least one enjoyable activity during non-meal times each day, preventing their lives from becoming entirely focused on swallowing difficulties. For specific issues, such as oral or pharyngeal residue, the patients were trained to apply problem-solving techniques to generate coping strategies. Their family members were guided to modify the home eating environment, including adjusting utensil types and table arrangements to reduce patient frustration. During the seventh session, a peer support group was organised to facilitate shared experiences, leveraging peer support to reduce feelings of stigma.

(5) Sessions 9–12 focused on enhancing hope levels and consolidating rehabilitation confidence. The therapist guided the patients to break down the long-term goal of restoring normal swallowing into a visualised rehabilitation milestone map, marking weekly improvements in surface electromyography parameters and water swallowing test grades. By using significant-other interview techniques, the therapist helped the patients recall past psychological resources for overcoming difficulties and assisted them in identifying potential positive gains from the illness, such as improvements in family relationships. The later sessions emphasised relapse prevention training, identifying high-risk situations like temporary declines in swallowing function after a common cold. The therapist and patients co-created a tangible emergency response card containing standardised coping procedures and emergency contact information. Prior to concluding the therapy, the patients completed a narrative writing exercise titled “My Rehabilitation Journey” and formulated a post-discharge self-management maintenance plan.

(6) Video feedback technology was utilised throughout the treatment process, with periodic recordings of the patients’ swallowing training sessions to counteract the cognitive bias of perceiving no improvement. Swallowing imagery training was conducted in conjunction with tDCS sessions, guiding the patients to vividly imagine safe and smooth eating processes to reinforce neural remodelling. All therapy sessions were meticulously documented using structured therapy records, detailing the session themes, the techniques applied and homework completion. The patients completed the STED self-help form daily to track cognitive, emotional and behavioural patterns. The personalised swallowing fear hierarchy and rehabilitation milestone map were archived as individual patient records. The therapy room was equipped with suction devices to manage severe aspiration events during exposure training. Adjustments were made for elderly patients, including reduced speech pace and the provision of large-print materials. Patients with mild cognitive impairment were allowed to have family members accompany them during certain sessions.

### Observed Indicators

#### General Data and Baseline Clinical Information

General data and baseline clinical information, including age, gender, BMI, NIHSS score, years of education, duration of dysphagia (days), cerebral infarction classification (anterior/posterior circulation), smoking history, alcohol consumption history and comorbidities, were collected by reviewing medical records. The duration of dysphagia was defined as the period from the date when swallowing abnormalities were first recorded in the medical records to the enrolment date, with the earliest documented description of dysphagia in the course of illness records, nursing records or rehabilitation assessment reports serving as the starting point.

#### Water Swallowing Test

Data on water swallowing test grades were extracted from the medical records at two time points: baseline (within 48 h prior to treatment initiation) and at the 4-week follow-up assessment. The grading criteria documented in the records were as follows: Grade I: The subject is able to swallow the water in one attempt without choking or any signs of dysphagia. Grade II: The subject requires two or more attempts to swallow the water without choking. Grade III: The subject is able to swallow the water in one attempt but with choking. Grade IV: The subject requires two or more attempts to swallow the water with choking. Grade V: The subject experiences frequent choking and is unable to swallow the full amount of water, indicating severe impairment of pharyngeal muscle movement. The grading criteria applied in this study were consistent with the standard water swallowing test protocol described by Kubota *et al* [9].

#### Surface Electromyography (sEMG) Parameters

sEMG parameters were extracted from the rehabilitation assessment records. According to the records, assessments were performed using the MyoMove-COW sEMG system (Shanghai Nuo Cheng Electric Co., Ltd., Medical Device Registration Certificate No. Hu Xie Zhun 20222070168) at baseline and after the 4-week treatment period. During the assessment, the patients were seated comfortably with their head in a neutral position. After the skin was prepared and cleaned, the recording electrode was placed over the infrahyoid muscle group, and the reference electrode was positioned on the dorsum of the hand or the mastoid process. The patients were instructed to perform a dry swallow, and the process was recorded three consecutive times, with the average value taken and recorded in the chart. The primary observation indicators included: (1) average amplitude, which reflects the average electrical potential intensity of muscle contraction during swallowing, and (2) maximum amplitude, which reflects the maximum muscle contraction strength during forceful swallowing. Higher values (μV) indicated better motor function and recruitment capability of the swallowing muscle groups.

#### Stigma Scale for Chronic Illness (SSCI) Scale

SSCI scores were obtained from the psychological assessment records at baseline and after the 4-week treatment period. The SSCI scale [13] used in the clinical assessments primarily included two measurement dimensions: external stigma (11 items) and internalised stigma (13 items). The scale employs a Likert 5-point scoring method, where each item is rated as follows: “Never” = 1 point, “Rarely” = 2 points, “Sometimes” = 3 points, “Often” = 4 points and “Always” = 5 points. The total score ranges from 24 to 120 points, with higher scores indicating stronger perceived stigma in patients.

#### Herth Hope Index (HHI) Scale

HHI scores were retrieved from the clinical records at baseline and after the 4-week treatment period. The HHI scale [14] used in the assessments primarily consisted of three dimensions: temporality and future (four items), positive readiness and expectancy (four items) and interconnectedness (four items). A Likert 4-point scoring method was employed, ranging from “Strongly Disagree” (1 point) to “Strongly Agree” (4 points), with higher scores indicating a greater level of hope in patients.

#### SAS and SDS scale

SAS and SDS scores were extracted from the patient-reported outcome measures documented in the medical charts at baseline and after the 4-week treatment period. The SAS [15] and SDS [16] scales used in the clinical evaluations consisted of 20 items. SAS includes five reverse-scored items, whereas SDS includes 10 reverse-scored items. After the raw scores are summed, the total is multiplied by 1.25 to convert it into a percentage-based score. Higher scores indicated more severe anxiety or depressive symptoms in patients.

#### NIHSS Scale

NIHSS [17] consists of 15 assessment items covering multiple dimensions, including level of consciousness, gaze, visual fields, facial palsy, limb motor function, ataxia, sensation and language. The total score ranges from 0 to 42, with higher scores indicating more severe neurological impairment.

### Statistical Processing

All data in this study were analysed using R statistical software (version 4.0.0, R Foundation for Statistical Computing, Vienna, Austria). Continuous variables were tested for normality by using the Kolmogorov–Smirnov test; measurement data conforming to a normal distribution were expressed as mean ± standard deviation. The baseline data and post-treatment efficacy indicators between the two groups were compared using paired t-tests or Wilcoxon signed-rank tests for continuous variables and McNemar’s test or Bowker’s test for categorical variables to control for the correlation within matched pairs due to the use of PSM. Comparisons before and after treatment within the same group were conducted using paired t-tests or Wilcoxon signed-rank tests. For instance, the ordinal data from the water swallowing test were compared using Wilcoxon signed-rank test. All tests were two-sided, and a *p*-value < 0.05 was considered statistically significant.

## Results

### Comparison of General Characteristics Between the Combination and Control Groups

No statistically significant differences were found between the two groups in terms of mean age, BMI, NIHSS score, years of education, duration of dysphagia, sex distribution, cerebral infarction classification, lifestyle history, types of comorbidities or the use of antipsychotic medication (*p* > 0.05, Table 1).

### Incidence of Adverse Events in the Two Groups

The adverse reactions that occurred during treatment were predominantly mild local skin reactions, including transient stinging sensation (combination group: 9.2% vs. control group: 8.0%), itching under the electrode (5.2% vs. 4.0%), skin erythema after treatment (13.8% vs. 12.6%) and mild burning sensation (2.9% vs. 1.7%). The systemic reactions included mild headache (6.9% vs. 5.7%), fatigue/somnolence (4.6% vs. 3.4%) and mild nausea (1.7% vs. 1.1%). All adverse reactions were mild to moderate (CTCAE grades 1 or 2), and they resolved spontaneously after rest, adjustment of stimulation intensity or cold compress. No statistically significant differences were observed in the incidence of adverse reactions between the two groups (*p* > 0.05). No serious adverse events such as skin burns or seizures occurred (Table 2).

**Table 2.** **Incidence of adverse events in the two groups**.

Type of adverse reaction		Combination group	Control group	Management	Severity grade
		(n = 174)	(n = 174)		(CTCAE 5.0)
Local skin reactions					
	Transient stinging sensation	16 (9.2%)	14 (8.0%)	Resolved spontaneously within 1 or 2 min after stimulation onset; no intervention required	Grade 1 (mild)
	Itching under the electrode	9 (5.2%)	7 (4.0%)	Resolved after cold compress or readjustment of electrode position	Grade 1 (mild)
	Skin erythema after treatment	24 (13.8%)	22 (12.6%)	Resolved spontaneously within 30–60 min after treatment cessation	Grade 1 (mild)
	Mild burning sensation	5 (2.9%)	3 (1.7%)	Resolved after reducing stimulation intensity (1.8 → 1.5 mA)	Grade 1–2 (mild to moderate)
Systemic reactions					
	Mild headache	12 (6.9%)	10 (5.7%)	Resolved after rest; no analgesics used	Grade 1 (mild)
	Fatigue/Somnolence	8 (4.6%)	6 (3.4%)	Resolved after 30 min of rest post-treatment	Grade 1 (mild)
	Mild nausea	3 (1.7%)	2 (1.1%)	Resolved spontaneously after rest	Grade 1 (mild)
Serious adverse events					
	Skin burns (blisters or ulceration)	0	0	—	—
	Seizures	0	0	—	—
	Study withdrawal due to adverse reactions	0	0	—	—

Note: CICTAE, Common Terminology Criteria for Adverse Events.

### Comparison of Water Swallowing Test Results Before and After Treatment

The water swallowing test results of both groups were compared before and after treatment. The overall distribution of the outcomes in the combination group after treatment was superior to that in the control group (*p* < 0.05, Table 3).

**Table 3.** **Comparison of water swallowing test results between the combination and control groups before and after treatment**.

Water swallowing test		Combination group	Control group	Z	*p*
		(n = 174)	(n = 174)		
Before treatment				−1.944	0.052
	Grade Ⅰ	0 (0)	0 (0)		
	Grade Ⅱ	13 (7.47)	20 (11.49)		
	Grade Ⅲ	99 (56.9)	105 (60.34)		
	Grade Ⅳ	50 (28.74)	45 (25.86)		
	Grade Ⅴ	12 (6.9)	4 (2.3)		
After treatment				−2.464	0.014
	Grade Ⅰ	28 (16.09)	26 (14.94)		
	Grade Ⅱ	120 (68.97)	98 (56.32)		
	Grade Ⅲ	24 (13.79)	39 (22.41)		
	Grade Ⅳ	2 (1.15)	9 (5.17)		
	Grade Ⅴ	0 (0)	2 (1.15)		

### Comparison of sEMG Parameters Before and After Treatment

Before treatment was applied, no statistically considerable differences were found in the sEMG parameters (average and maximum amplitudes) between the combination and control groups (*p* > 0.05). After treatment was applied, the measured values for the average and maximum amplitudes in the combination group were markedly higher than those in the control group (*p* < 0.05). Furthermore, compared with their respective pre-treatment measurements, the average and maximum amplitudes in both groups were markedly increased after treatment (*p* < 0.05, Table 4).

**Table 4.** **Comparison of surface electromyography parameters between the combination and control groups before and after treatment**.

Surface electromyography parameters		Combination group (n = 174)	Control group (n = 174)	t	*p*
Average amplitude (μV)					
	Before treatment	15.80 ± 2.67	16.25 ± 2.36	1.685	0.093
	After treatment	29.96 ± 2.87*	25.85 ± 2.33*	13.024	< 0.001
Maximum amplitude (μV)					
	Before treatment	29.55 ± 4.87	29.11 ± 5.99	0.752	0.453
	After treatment	58.72 ± 4.72*	51.48 ± 4.83*	12.856	< 0.001

Note: *p < 0.05 compared with pre-treatment within the same group.

### Comparison of Stigma Scores Before and After Treatment

Before treatment was administered, no statistically considerable differences were found in any of the stigma scores between the combination and control groups (*p* > 0.05). After treatment was administered, the internalised stigma scores and total SSCI scores in the combination group were markedly lower than those in the control group (*p* < 0.05). Compared with their respective pre-treatment measurements, both groups illustrated considerable reductions in the internalised stigma scores, external stigma scores and total SSCI scores after treatment (*p* < 0.05, Table 5).

**Table 5.** **Comparison of stigma scores between the combination and control groups before and after treatment**.

SSCI scale		Combination group (n = 174)	Control group (n = 174)	t	*p*
External stigma scores (point)					
	Before treatment	29.60 ± 4.75	30.44 ± 4.29	1.741	0.083
	After treatment	18.29 ± 2.83*	18.83 ± 2.45*	1.921	0.056
Internalised stigma scores (point)					
	Before treatment	44.99 ± 6.74	45.79 ± 6.58	1.119	0.264
	After treatment	22.46 ± 4.04*	26.61 ± 4.26*	−8.647	< 0.001
Total SSCI score (point)					
	Before treatment	74.59 ± 8.65	76.23 ± 8.11	−1.824	0.069
	After treatment	40.75 ± 5.12*	45.45 ± 4.73*	−8.231	< 0.001

Note: *p < 0.05 compared to pre-treatment within the same group. SSCI, Stigma Scale for Chronic Illness.

### Comparison of Hope Level Scores Before and After Treatment

Before treatment was applied, no statistically considerable differences were observed in any of the HHI subscale scores between the combination and control groups (*p* > 0.05). After treatment was applied, the scores for positive attitude toward reality and the future, positive actions taken and maintaining close relationships with others and the total HHI score were markedly higher in the combination group than in the control group, with statistically significant differences (*p* < 0.05). Furthermore, compared with their respective pre-treatment measurements, both groups showed considerable increases in the scores for positive attitude toward reality and the future, positive actions taken and maintaining close relationships with others and the total HHI score after treatment (*p* < 0.05, Table 6).

**Table 6.** **Comparison of hope level scores between the combination and control groups before and after treatment**.

HHI scale		Combination group (n = 174)	Control group (n = 174)	t	*p*
Temporality and future (point)					
	Before treatment	7.48 ± 2.11	7.84 ± 1.77	−1.724	0.086
	After treatment	11.69 ± 1.45*	11.22 ± 1.50*	2.876	0.003
Positive readiness and expectancy (point)					
	Before treatment	6.80 ± 1.54	7.06 ± 1.28	−1.713	0.088
	After treatment	11.39 ± 1.32*	10.81 ± 1.26*	3.994	< 0.001
Interconnectedness (point)					
	Before treatment	7.31 ± 1.71	7.59 ± 1.68	−1.541	0.124
	After treatment	9.77 ± 1.42*	9.31 ± 1.37*	2.981	0.003
Total HHI scores (point)					
	Before treatment	21.90 ± 4.17	22.49 ± 3.78	−1.383	0.168
	After treatment	32.84 ± 3.47*	31.34 ± 3.42*	3.892	< 0.001

Note: *p < 0.05 compared with pre-treatment within the same group. HHI, Herth Hope Index.

### Comparison of Depression and Anxiety Scores Before and After Treatment

Before treatment was administered, no statistically considerable differences were noted in the SAS and SDS scores between the combination and control groups (*p* > 0.05). After treatment was administered, the SAS and SDS scores in the combination group were significantly lower than those in the control group (*p* < 0.05). Compared with their respective pre-treatment measurements, both groups showed significant reductions in SAS and SDS scores after treatment (*p* < 0.05, Table 7).

**Table 7.** **Comparison of depression and anxiety scores between the combination and control groups before and after treatment**.

Items		Combination group (n = 174)	Control group (n = 174)	t	*p*
SAS scores (point)					
	Before treatment	55.69 ± 4.75	54.76 ± 6.25	1.563	0.119
	After treatment	38.30 ± 3.84*	41.60 ± 3.98*	−7.871	< 0.001
SDS scores (point)					
	Before treatment	55.93 ± 3.87	56.71 ± 5.50	−1.530	0.127
	After treatment	35.86 ± 3.00*	39.28 ± 3.56*	−8.943	< 0.001

Note: *p < 0.05 compared with pre-treatment within the same group. SAS, Self-Rating Anxiety Scale; SDS, Self-Rating Depression Scale.

## Discussion

The reconstruction of post-stroke swallowing function is a complex process involving the repair of cortical-medullary tracts and peripheral muscle coordination. The results of this study showed that patients who received CBT combined with tDCS in addition to routine rehabilitation demonstrated greater improvements in water swallowing test grades and sEMG parameters (average and maximum amplitudes) than those in the control group who received tDCS alone. Kumar *et al*. [18] noted that whilst tDCS is safe and can improve dietary intake in patients with acute to subacute stroke, its standalone application did not significantly reduce the risk of aspiration. This finding suggests that single-modality neuromodulation may be insufficient to fully reverse complex dysphagia. Clinical observations have indicated that patients often experience anticipatory fear and excessive tension due to choking during eating, which subsequently interferes with the coordinated contraction of normal swallowing muscles [19, 20]. In the present study, CBT combined with progressive behavioural experiments was applied to alleviate this abnormal muscular defensive state induced by the fear of choking. Following the intervention, the combination group exhibited a substantial increase in the mean and peak amplitudes on sEMG. This phenomenon may be attributed to the effective alleviation of local muscular hypertonia of psychological origin through cognitive restructuring, thereby allowing the swallowing muscles to achieve more effective recruitment and coordinated force generation under electrical stimulation, which manifested as a pronounced increase in the intensity of electromyographic activity [21, 22, 23].

This study found that all adverse reactions were mild to moderate (Common Terminology Criteria for Adverse Events (CTCAE) grade 1 or 2), without serious adverse events occurring. No statistically significant differences were observed in the incidence of adverse reactions between the two groups (*p* > 0.05), suggesting that the addition of CBT did not increase the risk of adverse effects associated with tDCS. All patients who experienced adverse reactions completed the full course of treatment, and no patient withdrew from the study due to adverse events. Furthermore, all patients in both groups completed the entire treatment protocol.

In addition to physiological impairments, the compromised self-image and social withdrawal resulting from dysphagia often act as invisible barriers during rehabilitation, directly affecting patients’ quality of life and confidence in reintegrating into the society. This study found that the combination group demonstrated significantly lower scores in internalised stigma and total SSCI scores after treatment, along with significantly higher scores across all dimensions of HHI than the control group. These findings align with observations from a mixed-method study by Choi and Kim [24], who reported that CBT significantly enhanced rehabilitation motivation and self-efficacy in patients with stroke. Patients with post-stroke dysphagia frequently experience severe self-devaluation due to issues such as drooling, nasogastric tube dependence or the need to consume pureed foods. This internalised stigma serves as a core barrier to social reintegration. Whilst conventional physical therapy can improve function, it often fails to address the deeply ingrained self-schema of being ‘disabled’ within the patient’s psyche [25, 26]. The combined treatment in this study utilised cognitive restructuring techniques to help patients dissociate dysphagia from their self-worth, thereby reducing internalised stigma. This improvement in psychological state aligns with the findings of Yue *et al*. [27], who confirmed that cognitive behavioural stress management can effectively promote neurological recovery and overall health status in patients with acute ischemic stroke by alleviating psychological distress. In the present study, objective electromyography data were integrated with rehabilitation milestones to assist patients in visually perceiving their functional progress. This cognitive behavioural intervention has been shown to remarkably enhance rehabilitation motivation in patients with stroke [24, 28].

In terms of emotional regulation, the SAS and SDS scores in the combination group were lower than those in the control group after the intervention. Whilst the evidence for the effectiveness of standalone psychological interventions in post-stroke rehabilitation remains inconclusive and of variable quality [29], the cognitive behavioural intervention employed in the present study specifically targeted dysphagia as a distinct stressor, potentially alleviating anxiety and depression by correcting negative automatic thoughts [30]. Although the control group received tDCS and showed improved swallowing ability, the degree of emotional improvement was relatively limited in the absence of targeted cognitive restructuring. However, this study is limited by the lack of direct neurophysiological and functional imaging assessments, leaving the exploration of the relevant neural mechanisms unsupported by objective evidence. Furthermore, the long-term efficacy of the intervention protocol could not be confirmed due to the limited observation period. Future research urgently needs to incorporate neuroimaging indicators and conduct multicentre prospective cohort studies to deeply investigate the precise mechanisms and long-term benefits of this combined modality. Additionally, this study employed a retrospective design, and the recording of adverse reactions relied on medical and nursing records, which may have resulted in undocumented mild discomforts. Future prospective studies should employ standardised adverse reaction questionnaires for systematic data collection to more comprehensively evaluate the safety of the combined intervention.

## Conclusions

The combination of CBT and tDCS was associated with improved swallowing motor function, reduced stigma and negative emotions and increased hope levels in patients, suggesting potential benefits at the physiological and psychosocial levels.

## Availability of Data and Materials

All experimental data included in this study can be obtained by contacting the corresponding author if needed.
